# Pathology for General Practitioners

**Published:** 1907-10-12

**Authors:** 


### Points in Pathology.

### PATHOLOGY FOR GENERAL PRACTITIONERS.

### The Use of the Freezing Microtome.

PERSONAL PREFERABLE TO LABORATORY INVESTIGATIONS.

Of late years the pursuit of pathology and bacteriology has come to be regarded as the special province of the professed pathologist and bacteriologist. As a result of this, there are nowadays but few practitioners who make any attempt at what may be called "clinical pathology," that is to say, the examination of blood, pus, sputum, and similar materials; the custom being to send such material to a research laboratory in London or elsewhere for examination and report. The disadvantages of such a course are various. In the first place it removes from the ken of the medical man an aspect of his practice which is of very great interest. In the second, to descend to a more worldly consideration, it entails extra expense to the practitioner or to his patient. Finally, it involves a delay of one or more days before the report can be received, a delay which it is always desirable and often most important to avoid. For instance, in cases of septicæmia it is necessary to determine the infecting organism with the utmost dispatch so that treatment by the corresponding serum or vaccine may be commenced at once. Again, in doubtful cases of typhoid fever, the presence or absence of Widal's reaction is a great aid to diagnosis; and both of these investigations can be easily carried out by the medical adviser with a minimum of apparatus, and in a very short time.

Several articles have recently appeared in The Hospital describing simple methods of examining blood, pus, and sputum, and of testing for Widal's reaction. In the present article an account will be given of an easy and rapid method of cutting sections of tumours and solid organs by means of the freezing microtome. No elaborate technique or costly apparatus is required, and a little practice will enable a medical man to pronounce judgment on a tumour within a quarter of an hour of its removal.

Quite apart from the interest which everyone will feel in being able to examine the new growths which he meets with in his practice, there is no doubt that a knowledge of this process will often be of great use to and bring considerable credit to the practitioner, as, for instance, in cases of ulcer of the lip and tongue, whose nature, whether innocent or malignant, it is almost impossible to determine from naked-eye appearances only. Some surgeons regard microscopical adjuncts to diagnosis as so important that, when operating on tumours of doubtful nature, they begin by removing a small fragment of the growth, and then suspend the progress of the operation for a few minutes until a section has been prepared, showing the nature of the growth and the consequent scope of the operation.

Two methods of procedure will be described, the choice between which depends on the time available. The first is exceedingly simple, and can be carried out in five to ten minutes, and is therefore used where an examination is required during the course of an operation. The second method undoubtedly gives better results than the previous one, but it is more complicated and cannot be carried out in so short a time. It is therefore advisable to become thoroughly acquainted with the first and easier method, which, after a little practice, will give very satisfactory specimens, before proceeding to the more difficult.

*Method* 1.—(*a*) Take a small piece of the tumour, roughly cubical in shape, measuring about a quarter-inch in each direction; this should, if possible, be taken from the margin of the growth, avoiding any part that is degenerated. Place the piece for a few seconds in a small bottle containing a 5 per-cent. solution of formalin in distilled water.

(*b*)—Transfer the specimen to the plate of a freezing microtome; by means of a camel's-hair brush surround it with a little of the formalin solution, and freeze as quickly as possible. It is well at first not to put too much of the formalin solution on the microtome, as this delays freezing, but as soon as solidification begins a little more may be painted on with the brush, until the specimen is firmly fixed to the plate.

(*c*) As soon as the block is sufficiently hard proceed to cut the sections. If the freezing takes place very slowly, it may be that the block is too thick. This can be remedied by cutting thin slices off the surface until a harder part is reached. Or, again, the small hole through which the ether escapes below the plate often becomes clogged with dust, so that a vigorous use of the bellows produces no effect.

(*d*) Place the sections in a glass dish containing some distilled water. It is convenient to cut about ten sections at a time, and to brush the accumulated mass off the razor into the water, separating the sections by gently stirring with the brush.

(*e*) Select a good section and transfer it by means of a section lifter and needle to a clean glass slide. The slides must be free from grease, or water will not flow evenly over them.

(*f*) Place on the section as it lies on the slides one drop of Loeffler's methylene blue.

(*g*) After ten seconds drain off as much stain as possible by means of blotting paper; pour on a few drops of distilled water; drain this off with blotting paper and repeat this washing once or twice to get rid of the excess of stain.

(*h*) Remove the water with blotting paper; place on the section a drop of glycerine, and cover with a cover-glass. The glycerine serves to clear the specimen, and renders it more transparent. The clearing effect of glycerine cannot be compared with that obtained by using alcohol and xylol, but sections stained with methylene blue cannot be satisfactorily treated with alcohol as this reagent dissolves much of the stain.

*Method* 2.—The sections are to be stained with hæmatoxylin and eosin, dehydrated with alcohol, and mounted in canada balsam.

(*a*) Remove a piece of the tumour as in method 1. Place it in 5 per cent. formalin for half an hour to 12 hours, according to the time available. This treatment, though not absolutely necessary, greatly facilitates the subsequent cutting and staining; and, of course, it is equally advantageous in Method 1, if time allows. (*b*), (*c*), (*d*) as in Method 1. (*e*) Select several of the better sections, and transfer to another glass dish containing distilled water, allow them to remain in this for a minute or two in order to wash out the formalin. (*f*) Stain the sections in a 5-per cent. solution of Ehrlich's hæmatoxylin in distilled water. They must be left in this staining solution for 5 to 15 minutes; the time required varies with the thickness of the section, and some practice is necessary to judge what is a sufficient depth of stain. (*g*) Wash the sections in distilled water for a few seconds to get rid of excess of stain. (*h*) Transfer the sections to tap-water, when they will be seen gradually to become deep blue in colour, and they must not be removed from the tap-water until the change of colour is complete. This reaction which depends on the alkalinity of tap water, can be hastened by warming the water to 40° C.; or, if the change be very slow, by adding one or two drops of ammonia to the water, an expedient to be avoided if possible, as it may damage the sections. (The various transferences of the sections required in (*e*), (*f*), (*g*), (*h*) may be conveniently carried out by lifting them on the end of the camel's-hair brush. This takes much less time than removing them with a section lifter, and does not seem to damage the specimens.) (*i*) By means of a section lifter and needle place one of the sections on a clean slide; drain off the water and press the section firmly on the slide with blotting paper soaked in methylated spirit. If the blotting paper be dry, the section will stick to it, and will come off the slide. (*j*) Flood the slide two or three times with methylated spirit to remove the water from the section. (*k*) Flood the slide once or twice with absolute alcohol to remove the methylated spirit. (*l*) Counterstain the section for 5 to 15 seconds in a few drops of a saturated solution of eosin in absolute alcohol. (*m*) Wash off the excess of stain with absolute alcohol. (*n*) Pour on xylol to clear the section. Xylol can mix with and take the place of absolute alcohol, but it is not miscible with water; consequently if the section has not been properly dehydrated, a white cloud will form when the xylol is poured on. This may disappear on adding more xylol; if it does not, the section must again be treated with absolute alcohol. When the section is properly cleared it will be quite transparent and almost invisible when held against a dark background. (*o*) Remove excess of xylol with blotting paper, place on the section a drop or two af canada balsam and cover. If correctly stained the nuclei will appear dark blue, and the plasma and interstitial substance pink. Frozen sections always stain rather diffusely, but this diffuseness is less marked if a weak solution of hæmatoxylin (such as is recommended above) be used.

